# Comparing the Effects of Glyphosate and Mycotoxins in the Human Follicular Microenvironment: An Exploratory Exposome Study

**DOI:** 10.3390/biomedicines14051081

**Published:** 2026-05-09

**Authors:** Apolka Szentirmay, Márkó Unicsovics, Eszter Ruff, Bernadett Csókay, Katalin Sára-Popovics, Dóra Holéci, Tímea Buzder, Miklós Sipos, Attila Martonos, Attila Sajgó, Natália Szeőcs, György Nagyéri, Levente Sára, Zsuzsanna Szőke

**Affiliations:** 1 Department of Obstetrics and Gynecology, Semmelweis University, 1088 Budapest, Hungary; szentirmay.apolka.kinga@semmelweis.hu (A.S.); unicsovics.marko@semmelweis.hu (M.U.); eszteruff@gmail.com (E.R.); cspupi@gmail.com (B.C.); 2 Department of Animal Biotechnology, Institute of Genetics and Biotechnology, Hungarian University of Agriculture and Life Sciences, 2100 Gödöllő, Hungary; katalinsarapopovics@gmail.com (K.S.-P.); holeci.dora@uni-mate.hu (D.H.); nagyeri.gyorgy@uni-mate.hu (G.N.); ferenczine.szoke.zsuzsanna@uni-mate.hu (Z.S.); 3 Department of Chemistry, Institute of Mathematics and Basic Science, Hungarian University of Agriculture and Life Sciences, 2100 Gödöllő, Hungary; 4 Central of Assisted Reproduction, Semmelweis University, 1097 Budapest, Hungary; buzder.timea@semmelweis.hu (T.B.);; 5 Department of Microbiology and Applied Biotechnology, Institute of Genetics and Biotechnology, Hungarian University of Agriculture and Life Sciences, 2100 Gödöllő, Hungary; natalia.sz@cebiosys.com; 6 Central European Biosystems Ltd., 1044 Budapest, Hungary; 7 Szentágothai Research Center and Department of Neurobiology, University of Pécs, 7624 Pécs, Hungary

**Keywords:** endocrine disruptors, glyphosate, follicular fluid, oxidative stress, mycotoxins, gonadal hormones

## Abstract

**Background**: Exposure to anthropogenic and/or natural (e.g., herbicides or mycotoxins) endocrine-disrupting chemicals (EDCs) has been linked to several reproductive disorders. Glyphosate (GLY), a common agricultural agent, is a potential element of the exposome that bioaccumulates and has potential endocrine and oxidative stress-related effects. However, data on its presence in the human ovarian microenvironment remain limited. Our study examined GLY levels in follicular fluid (ff) and serum and their relationships with oxidative stress markers, reproductive hormones, and stress hormones in women undergoing in vitro fertilization (IVF). **Methods**: 50 women undergoing controlled ovarian stimulation participated. Serum and ff samples were routinely collected during oocyte retrieval. GLY, related hormones (e.g., cortisol, estradiol-E2, anti-Müllerian hormone-AMH, and melatonin-MT), an oxidative stress marker malondialdehyde (MDA), antioxidant enzyme activities, total antioxidant capacity, and co-occurring natural pollutant mycotoxin levels were measured. Relationships between GLY levels and these mediators were assessed using correlation and regression analyses. **Results**: GLY was detected in both serum and ff at similar concentrations (0.038 ± 0.006 ng/mL vs. 0.045 ± 0.006 ng/mL; *p* = 0.414). Follicular GLY levels showed a positive association with MDA (Spearman’s r = 0.4487, *p* < 0.001), explaining 28.6% of the variability in follicular MDA. Serum GLY was positively associated with serum (β = 40.26, *p* = 0.0058) and follicular E2 (r = 0.29, *p* = 0.042). Serum GLY levels were negatively correlated with cortisol (β = −0.0188, *p* = 0.020). A slight correlation between follicular GLY and MT was observed (*p* = 0.03). No associations were found between GLY levels and age, body mass index, AMH, the recombinant gonadotropin dose used, antioxidant enzyme activities, follicle count, oocyte yield, or embryo viability. **Conclusions**: This study might be the first to demonstrate the presence of GLY of exposome in human ff, indicating that environmental exposure to GLY may reach the oocyte microenvironment. The correlation with lipid peroxidation suggests GLY could contribute to follicular oxidative stress. The associations with E2 and cortisol point to potential endocrine-disrupting effects. While no direct impact on *IVF* outcomes was observed, findings suggest low-level exposure to GLY could influence ovarian physiology through specific biochemical mechanisms.

## 1. Introduction

The burden of infertility affects 10–15% of couples worldwide who are of reproductive age, and one-third of cases remain unexplained [1]. The increasing focus on environmental exposure to various natural and/or anthropogenic pollutants (the exposome) has heightened attention to endocrine-disrupting chemicals (EDCs), which already pose a global threat to environmental, animal, and human health. These substances [2] are defined as exogenous compounds that can interfere with many aspects of hormone actions. To date, more than 1000 agents have been identified as endocrine disruptors, including pesticides, fungicides, mycotoxins, industrial chemicals, plasticizers, nonylphenols, metals, pharmaceuticals, and phytoestrogens. The primary route of animal and human exposure to EDCs is through dietary intake (contaminated drinking water, grains, and processed products), with inhalation and dermal contact also contributing [3]. To date, due to industrialization, human activities, environmental pollution, and/or global warming, human populations have to face different patterns of acting exposome, exposures to such pollutants. In our study, we highlight the pesticide glyphosate (GLY) as a major co-occurring pollutant and examine its potential association with fertility.

GLY is one of the most widely used agricultural herbicides globally [4]. It was originally designed to affect only plants and microorganisms by targeting the shikimate pathway—a metabolic route absent in vertebrates—thus having theoretically minimal toxicity to mammals [4,5]. The widespread use and escalating use of GLY-based herbicides (GBHs)—especially formulations like Roundup of Monsanto—have led to global environmental contamination and extensive human exposure [6]. GLY and its metabolites—particularly the aminomethylphosphonic acid (AMPA), as a primary metabolite—are now detectable in various animal and human matrices, such as blood, urine, and breast milk. Detection rates vary across populations and samples, ranging from 70% to 99% [4,7,8]. GLY and AMPA have been shown to cause no or minimal harm to non-target organisms, but recent studies have raised concerns about their effects, which may lead to various diseases [9]. Growing evidence suggests that, in addition to its carcinogenic potential, GLY, GHBs, and their metabolites may function as EDCs, thereby modulating the predominantly endocrine-driven reproductive system [10,11]. In numerous studies, GLY has been identified as an EDC agent that can affect hormone receptor activity or disrupt hormone transport [8]. It may alter estrogen and androgen synthesis, hormone receptor formation, and hormone receptor activity [12]. The harmful effects on the reproductive system are detectable in both males and females. At every level of the hypothalamic–pituitary–gonadal axis, including the hypothalamus, anterior pituitary gland, ovaries, and testes, GLY has been shown to have documented effects [11,13]. GLY can interact, *inter alia*, with estrogen receptors (ERα and ERβ), mimic the effects of 17β-estradiol (E2), and promote estrogen receptor phosphorylation and transcriptional activity [11,14]. In the female reproductive system, where the follicle represents a stable microenvironment for the oocytes, these effects are highly relevant [11]. Regarding the oocyte maturation and development, the content of follicular fluid (ff) in tertiary follicles surrounding the cumulus cells is essential. Hormones, proteins, free fatty acids, and other bioactive molecules secreted by theca and the granulosa cells can affect the quality of the oocyte. Variations in the biochemical profile of ff can significantly reduce fertility [15].

The generation of reactive oxygen species (ROS) has a critical role in oocyte maturation, both in vivo and in vitro. Antioxidant agents help regulate excessive oxidative stress caused by ROS, which is important for normal cellular function. Alterations in oocyte functions, arrested meiotic progression, and, hence, poor reproductive outcomes can all be caused by an imbalance between antioxidants and ROS [16,17]. Malondialdehyde (MDA) is a reactive end-product, generated through the peroxidation of polyunsaturated fatty acids in biological membranes caused by ROS [18,19]. Recently, antioxidant supplementation has become a focus of attention, as it may improve oocyte and sperm quality and increase the rate of spontaneous pregnancy in an in vitro fertilization (*IVF*).

It has been demonstrated that GLY and its metabolites alter ROS levels [20,21]. Their effects on antioxidant enzyme activity may further alter the oocyte’s oxidative status, thereby impairing reproductive outcomes. To mitigate adverse reproductive outcomes, it is important to understand the role of environmental contaminants, such as the currently focused GLY, other pesticides, or natural expositors (e.g., mycotoxins), in the ff.

Mycotoxins are common contaminants of food and feed produced by fungi and are increasingly recognized as co-occurring endocrine-disrupting pollutants. Compounds can interfere with hormone signaling, including estrogenic and steroid pathways, contributing to reproductive and metabolic effects in humans. Importantly, human exposure typically involves complex mixtures of mycotoxins alongside other endocrine-disrupting chemicals (e.g., pesticides, plasticizers), raising concerns about additive or synergistic effects at low doses. Such combined exposures are difficult to capture using single-chemical risk assessment frameworks, yet they are more representative of real-world conditions. This has led to growing interest in incorporating mycotoxins into broader exposome and mixture-toxicity approaches in public health research [22,23].

Present studies examining the correlation between exposure to GLY and human fertility are limited, biased, and provide inconsistent results due to the broad spectrum of causes of reproductive disorders and the challenges in exposure assessment. Our study, therefore, aims to examine in context, exposome element GLY levels in vivo and their potential effects on human fertility, with particular emphasis on their presence in ff. The simultaneous detection of the most common mycotoxins and their metabolites provides a more complex picture of the multiple environmental toxic effects on follicles and may also help with a more accurate assessment of GLY effects on oxidative stress.

## 2. Materials and Methods

Infertile patients undergoing controlled gonadotropin stimulation in May 2025 were enrolled in this study. After receiving comprehensive verbal and written information and providing written informed consent approved by the regional ethics committee (SE-RKEB 86/2023), 50 patients participated. Serum hormone levels (follicle-stimulating hormone—FSH, luteinizing hormone—LH, and E2) were measured on cycle days 2–4 and 12, while anti-Müllerian hormone (AMH) had been assessed in previous cycles during diagnostic evaluation. Prior to oocyte retrieval, transvaginal ultrasonography was performed to accurately measure visible follicles. Oocyte retrieval as a routine procedure was performed at the Center for Assisted Reproduction, Department of Obstetrics and Gynecology, Semmelweis University, Budapest. On the day of retrieval, blood samples were collected to determine serum mycotoxin levels as described below, and transvaginal follicular aspiration was performed under surgical conditions. After oocyte isolation, ff was collected and centrifuged. Samples visibly contaminated with blood were excluded. To minimize procedural intervention and the risk of blood contamination, ff of a patient from all follicles (from both ovaries) after the harvest were pooled rather than collected individually [24]. All serum and ff samples were stored at −70 °C until use.

### 2.1. Laboratory Analyses

#### Measurement of GLY, Mycotoxins, and Hormone Levels in Serum and Follicular Fluid Samples

To quantify GLY levels in the samples, we used the Eurofins Abraxis Glyphosate Plate ELISA Kit (PN 500205). Although the assay protocol is mainly designed for analyzing human serum samples, we also applied it to ff in our study. Ff is considered a transudate or exudate of serum; thus, the procedure was adaptable to this biological matrix. During sample preparation, 500 µL of the sample was pipetted into a Millipore Amicon Ultra 0.5 mL 10k centrifugal filter (Cat. No. UFC501096, Merck KGaA, Darmstadt, Germany). For potentially turbid samples, the volume was split between two filters to ensure enough supernatant was collected for subsequent steps. Next, the samples were centrifuged at 8000× *g* for 15 min. After centrifugation, 300 µL of the supernatant was transferred to a new microcentrifuge tube, and 200 µL of ethyl acetate was added. The samples were vortexed for 30 s and centrifuged again at 8000× *g* for 3 min. The lower aqueous phase was then transferred to a new tube. During derivatization, because the optimal 250 µL sample volume is not always achievable after extraction, we used half of the recommended reagent volume (125 µL sample, 500 µL assay buffer, and 50 µL diluted derivatization reagent). The basic dilution process for the derivatization reagent (with 3.5 mL of diluent) remained unchanged. To prevent potential degradation, measurements were performed as soon as possible after extraction, and samples were stored in plastic microcentrifuge tubes for no more than 1 day before analysis. The average recovery rate of the method, validated on serum samples, was 91%, while in our study, the recovery rate for ff ranged from 82.5% to 93.2%. The cross-reactivity of the kit with glycine, AMPA, and glufosinate was negligible.

Mycotoxins were quantified in plasma and ff samples according to previously published methods [2,6]. Zearalenone (ZEA), Ochratoxin A (OTA), Fumonisin B1 (FB1), Deoxynivalenol (DON), T2/HT2 toxin, total aflatoxins (Afs: B1, B2, G1, G2), and aflatoxin M1 (AM1) were determined using immunoassays, while alpha-Zearalenol (α-ZOL) was analyzed by GC-MS [23].

Steroid hormones in serum and ff were measured using commercial ELISA kits for E2 (Cat No: DNOV003, NovaTec Immundiagnostica, Dietzenbach, Germany) and progesterone-P4 (Cat No: DNOV006, NovaTec, Dietzenbach, Germany) [25]. Cortisol concentrations were also measured by immunoassay (Cat No: DNOV001, Gold Standard Diagnostic, Dietzenbach, Germany) according to the manufacturer’s protocol [26].

The concentrations of dihydrotestosterone (DHT) in serum and ff samples were measured using a competitive ELISA kit (EIA-5761, DRG Instruments GmbH, Marburg, Germany) according to the manufacturer’s instructions. For ff samples, preliminary optimization with the provided sample dilution buffer determined that a 20-fold dilution was optimal, and all ff measurements were conducted using this dilution. The assay’s limit of detection (LOD) was 6 pg/mL.

Serum and ff MT levels were measured using a Human MT ELISA Kit (Catalog No.: RE2184H, Reed Biotech Ltd., Wuhan, China), which functions on a competitive enzyme immunoassay principle. The process was strictly followed by the manufacturer’s instructions. The assay’s sensitivity was 9.38 pg/mL, with a detection range of 15.63–1000 pg/mL.

All measurements mentioned above were performed in triplicate. Absorbance, optical density (OD), was recorded at 450 nm with a reference wavelength of 630 nm using a Thermo Multiskan^TM^ FC microplate reader (Waltham, MA, USA) and SkanIt RE software (version 6.1.1.7).

Serum AMH levels were determined using the Beckman Coulter Access 2 Immunoassay System (California, USA) with a chemiluminescent immunoassay (CLIA). The Access AMH Advanced test (Cat. No. B13127, Beckman Coulter, Brea, CA, USA) was performed according to the manufacturer’s instructions, and samples were analyzed in duplicate [25].

Total protein concentration of the samples was measured at 280 nm using a NanoDrop spectrophotometer (Thermo Fisher Scientific, Waltham, MA, USA). The ‘Protein A280’ software setting was used, applying the BSA mass extinction coefficient for concentration calculations. Baseline correction was performed at 340 nm, and 0.01 M PBS was used as the blank. All measurements were conducted in quintuplicate.

### 2.2. Antioxidant Enzyme and Total Antioxidant Capacity Analyses

Superoxide-dismutase (SOD) activity in ff was determined using the Invitrogen Superoxide Dismutase Colorimetric Activity Kit (Catalog Number EIASODC). Ff samples were diluted (1:5) in 1X Assay Buffer, and 10 μL of diluted sample was added to a 96-well plate. Following the addition of 1X Substrate and Xanthine Oxidase, the reaction was incubated at RT for 20 min. Absorbance was measured at 450 nm. When necessary, pre–Xanthine Oxidase absorbance values were subtracted to correct for background coloration. Total SOD activity was calculated according to the manufacturer’s instructions.

Catalase (CAT) activity was assessed using the Catalase Colorimetric Activity Kit (Catalog Number EIACATC, Invitrogen, Carlsbad, CA, USA). Ff samples were lysed in 1X Assay Buffer, and the supernatant was diluted accordingly. Samples and standards were incubated with Hydrogen Peroxide Reagent, followed by Substrate and 1X horseradish peroxidase solution. After incubation, absorbance was measured at 560 nm, and CAT activity was calculated from a standard curve of known CAT concentrations.

TAOC in ff was evaluated using the Total Antioxidant Capacity Colorimetric Assay Kit (ABTS, Enzyme Method) (Catalog No. EEA023, Invitrogen). Samples and Trolox standards were incubated with peroxidase application solution and ABTS working solution. After 6 min at room temperature, absorbance was recorded at 414 nm. TAOC values were calculated based on the Trolox standard curve [24].

All absorbance measurements were performed using a Thermo Multiskan^TM^ FC microplate reader (Waltham, MA, USA) with SkanIt RE software (version 6.1.1.7).

### 2.3. Follicular Fluid MDA Assay

Lipid peroxidation was assessed by measuring MDA levels in ff with the Thiobarbituric Acid Reactive Substances Assay. The measurements were performed using a Lipid Peroxidation (MDA) Assay Kit (MAK085, Sigma-Aldrich, Merck, Darmstadt, Germany) and followed the manufacturer’s instructions. In this method, MDA in the ff reacts with thiobarbituric acid (TBA) to form an MDA-TBA complex, which is quantified by colorimetric analysis. A 0.1 mol/L MDA standard was prepared, along with serial dilutions to generate a standard curve. Both the samples and standards were pipetted into a 96-well plate, and absorbance was measured using a Thermo Multiskan^TM^ FC (Waltham, MA, USA) with SkanIt RE software (version 6.1.1.7). Readings at 532 nm were taken, and the absorbance of the blank (ultra-pure water) was subtracted to correct for background interference [26].

### 2.4. Statistical Analysis

Statistical analyses were performed using statistical software (GraphPad Prism, version 11.0.0 (93), GraphPad Software LLC, 2026). Data distribution was assessed with the Shapiro–Wilk, D’Agostino–Pearson, and Anderson–Darling tests. When necessary, a square-root transformation was applied to achieve normality and stabilize variances. Continuous variables are presented as mean ± SEM or median, as appropriate. Group comparisons were conducted using the Mann–Whitney U test and the paired *t*-test for non-normally distributed data. Correlations were evaluated using Pearson’s or Spearman’s coefficients, depending on distribution. Associations between GLY levels and clinical or biochemical parameters were analyzed by simple linear regression. Model assumptions were verified by residual diagnostics. Regression coefficients (β) are reported with 95% confidence intervals (CIs), and R^2^ values indicate the proportion of variance explained. A two-sided *p*-value < 0.05 was considered statistically significant (*n* = 50). For exploratory analyses involving multiple comparisons, the Benjamini–Hochberg false discovery rate (FDR) procedure was used to control for the expected proportion of potential false-positive findings.

## 3. Results

### 3.1. Comparison of Serum and Follicular Fluid GLY Concentrations

**Figure 1:**
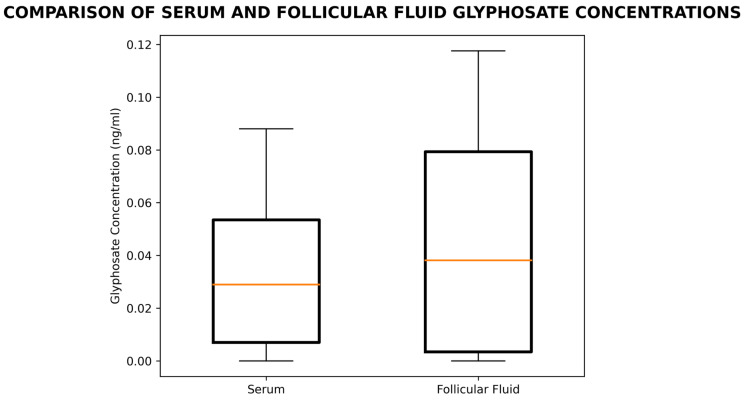
Comparison of serum and follicular fluid GLY concentrations. Each box represents the interquartile range, with the median shown as a horizontal line; whiskers indicate the 5th and 95th percentiles (*n* = 50 per group).

GLY was found in both serum and ff. The levels in ff were not significantly different from those in serum. The mean ± SEM concentrations in ng/mL were 0.045 ± 0.006 in ff vs. 0.038 ± 0.006 in serum. (Figure 1).

**Figure A1:**
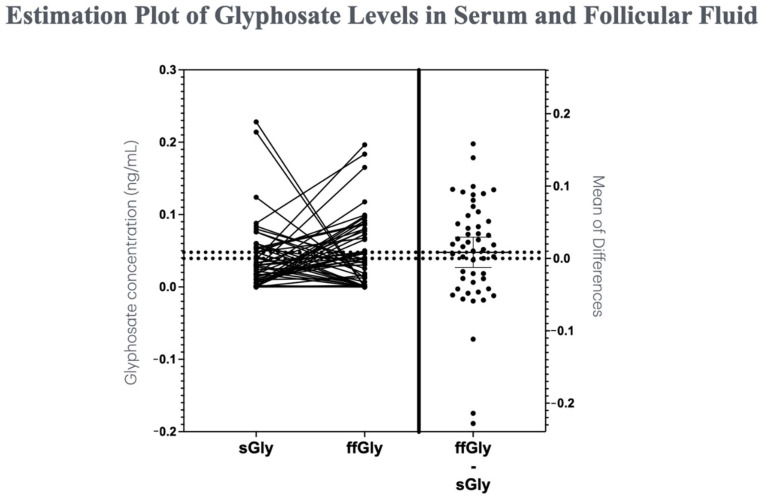
Comparison of serum and follicular fluid GLY concentrations using a paired *t*-test on a nonparametric dataset. On the left, each dot represents the patient’s GLY concentration (ng/mL), with lines connecting paired serum and ff values. On the right, the mean difference is shown. (*n* = 50 per group).

Median concentrations were 0.029 ng/mL in serum and 0.038 ng/mL in ff. The difference was not statistically significant (U = 1131, *p* = 0.414), suggesting that GLY levels in serum and ff were comparable (Figure A1). There was no correlation between serum and ff GLY concentrations. Likewise, there was no correlation between GLY in the ff and the concentration of the ff protein.

### 3.2. Association Between ffGLY Exposure and ffMDA Concentrations

Since GLY concentrations were not normally distributed, Spearman correlation was used, revealing a strong positive link between follicular MDA and GLY levels (r = 0.4487, *p* < 0.001). To achieve normality and stabilize variance, a square root transformation was applied to both the ffGLY and ffMDA datasets. After the transformation, all normality tests (Shapiro–Wilk, D’Agostino–Pearson, and Anderson–Darling) yielded non-significant results (*p* > 0.3), confirming that the residuals satisfied the assumption of normality.

**Figure 2:**
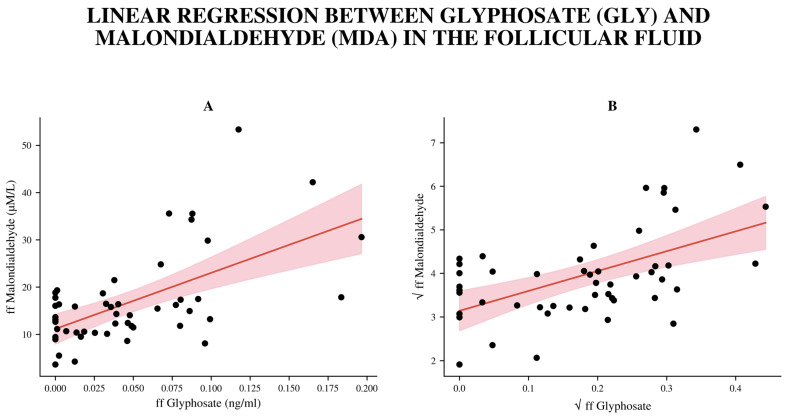
Illustrates the relationship between ffMDA and ffGLY concentrations (**A**) and after a square root transformation (**B**). There is a positive correlation: as ffGLY increases, ffMDA generally rises. Black points represent the observed data, while the red lines show the fitted regression line. The shaded light-red areas mark the 95% confidence interval for the predicted mean.

A simple linear regression model was fitted to assess the relationship between √ffMDA (dependent, square-root-transformed variable) and √ffGLY (independent square-root-transformed variable). The regression model was statistically significant (F(1.48) = 19.25, *p* < 0.0001), with a coefficient of determination (R^2^) of 0.2863, indicating that approximately 28.6% of the variance in ffMDA was explained by variation in ffGLY. The slope coefficient (β_1_ = 4.560, 95% CI (2.47–6.65)) was highly significant (*p* < 0.0001) after FDR correction, suggesting a strong positive association between ffGLY and ffMDA concentrations (Figure 2). The intercept of transformed data (β_0_ = 3.139, 95% CI (2.68–3.60)) remained significant. Residual diagnostics confirmed model assumptions: residuals were symmetrically distributed, independent, and had constant variance across fitted values. No multicollinearity issues were detected. ffMDA levels did not show correlations with age, BMI, or gonadotropin treatment.

### 3.3. Association Between GLY Exposure and Serum and ff E2 Concentrations

**Figure 3:**
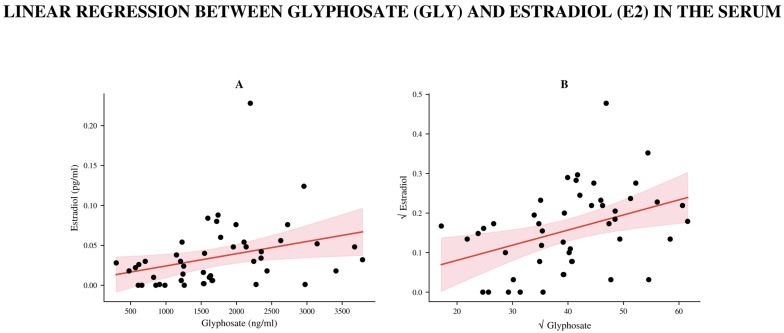
Linear regression between GLY and estradiol concentrations in the serum. Because the raw data (**A**) were not normally distributed, serum GLY and estradiol values were square-root transformed for appropriate parametric analysis (**B**). The scatter plot shows the relationship between GLY and E2 (*n* = 48). The red line represents the least-squares linear regression, and the light red area indicates the 95% confidence interval of the regression line.

A notable positive link was found between serum GLY levels and E2 (Spearman r = 0.45, 95% CI: 0.18–0.66, *p* = 0.0013; *n* = 48, Figure 3). Since the raw data were not normally distributed, both serum GLY and E2 values were square-root transformed before parametric analysis to better approximate normality. Linear regression on the transformed data showed that serum GLY was a significant predictor of E2 levels (β = 40.26, 95% CI: 12.25–68.28, *p* = 0.0058), accounting for 15.4% of the variation in estradiol levels (R^2^ = 0.154). Residual diagnostics confirmed that the normality assumptions were met after transformation.

**Figure 4:**
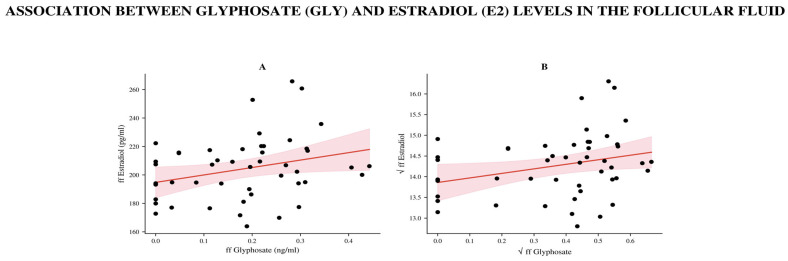
Scatter plot showing the relationship between ffGly and free ffE2 concentrations. The raw data (**A**) were square-root-transformed to avoid statistical errors (**B**). Each point represents one sample (*n* = 49). The solid red line shows the least-squares fit (β = 0.00154, *p* = 0.047), and the light red area represents the 95% confidence interval of the regression.

Similarly, analysis of ff samples showed a weak but statistically significant positive correlation between ffGly and ffE2 levels (Spearman r = 0.29, 95% CI: 0.0017–0.5348, *p* = 0.042; *n* = 49, Figure 4).

After square root transformation, linear regression yielded β = +0.00154 (95% CI (0.00002, 0.00307), *p* = 0.047, R^2^ = 0.081, *n* = 49), indicating that an increase in ffE2 was associated with a slight increase in ffGLY levels. Residuals were normally distributed (*p* > 0.1 for all tests). This finding is consistent with the Spearman correlation (r = 0.291, *p* = 0.043). However, these correlations lost their significance after FDR correction.

Overall, these results demonstrate the same trends: total GLY exposure is positively associated with serum E2 concentration, and the relationship between their free fractions is also weakly positive in the ff.

A slight positive correlation was also detectable between follicular GLY and P4 concentrations; however, after linear regression, the significant relationship disappeared.

### 3.4. Association with Biophysical, Antioxidant, Mycotoxins, and Other Reproductive Parameters

We found no association between GLY levels measured in serum or ff and age, body mass index (BMI), AMH, recombinant gonadotropin treatment, testosterone, DHT, or P4. Based on the 50 samples examined, we found no correlation between follicular and serum GLY levels and the number of follicles, number of dominant follicles, number of oocytes, and number of usable embryos. We also found no correlation between the mycotoxins tested and GLY. As in the previous results, there was no correlation between GLY and SOD, CAT, or TAOC levels in the ff (Table A1).

We found a positive correlation between follicular GLY and MT concentrations (*p* = 0.03). After applying the square-root transformation to the data, the linear regression analysis demonstrated a positive trend but did not reach statistical significance (*p* = 0.09).

**Figure 5:**
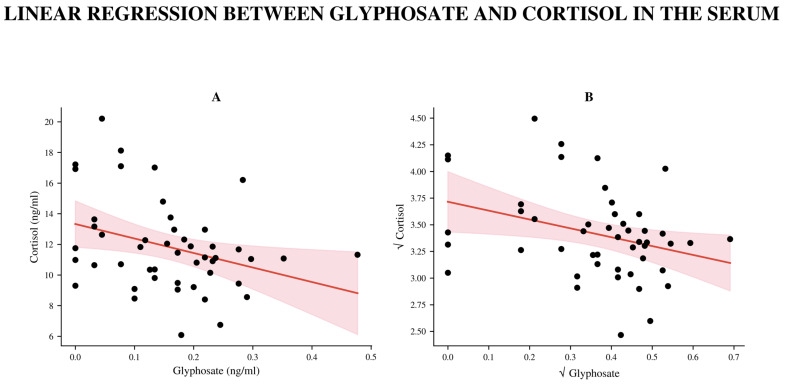
Linear regression analysis of the association between serum GLY and cortisol concentrations. (**A**) Linear regression between serum GLY concentration (ng/mL) and serum cortisol concentration (ng/mL) using the original data. Since the raw data did not show a normal distribution, we applied a square-root transformation. (**B**) The red line indicates the fitted regression line, and the light red area represents the 95% confidence interval of the regression.

We observed a stronger negative correlation between GLY and serum cortisol levels, with a significant linear relationship persisting even after transforming the data to achieve normality (β = −0.0188, 95% CI −0.0344 to −0.0031; R^2^ = 0.112; *p* = 0.020; *n* = 48, Figure 5). The significance disappeared after FDR correction. There was no correlation between GLY and cortisol levels in the ff (Table A1).

**Figure 6:**
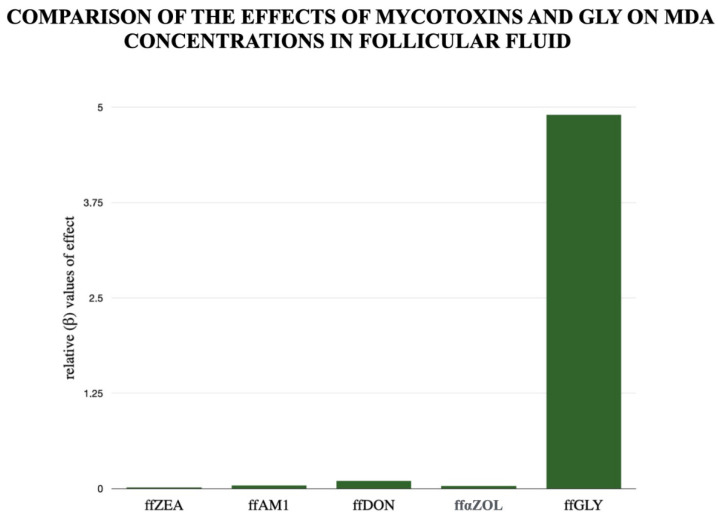
Comparison of the Effects of Mycotoxins and GLY on MDA Concentrations in Ff. ffZEA: follicular Zearalenon, ffAM1: follicular Aflatoxin M1, ffDON: follicular Deoxinyvalenol, ff αZOL: follicular alpha-Zearalenol, ffGLY: follicular GLY. Relative effect (β) values were calculated from standardized regression coefficients derived from Pareto-based linear regression models. Standardization enabled direct comparison of the relative contributions of mycotoxins and GLY treatments to changes in MDA concentrations.

We have also examined the potency of relevant mycotoxins (compared with GLY) in inducing oxidative stress, as reflected in MDA levels, as shown in Figure 6. Our research team has previously presented the role of certain mycotoxins in oxidative stress during human folliculogenesis [26]. The mycotoxin potencies shown in Figure 6 clearly demonstrate the importance of GLY as a control for predicting MDA concentration. A Pareto analysis shows the relative potency (β-value) of the mycotoxins and GLY. The figure clearly shows the extent to which concentrations measured under physiological conditions correlate with MDA levels, and thus their relative importance in inducing oxidative stress. GLY had increased potency; mycotoxins could not show that impact.

## 4. Discussion

Human populations must face a growing burden of environmental stressors collectively described within the field of exposome, the totality of external–internal exposures in an individual (over a lifetime) [27]. Increasing industrialization, agriculture, and urbanization have amplified environmental load, leading to widespread pollution of air, water, soil, and food or feed. These pollutants include endocrine-disrupting, persistent chemicals, many of which accumulate/present in ecosystems and human tissues as well [28]. Within this framework, both anthropogenic and natural pollutants can be relevant. GLY has gained particular attention due to its extensive global use and environmental persistence, making it a highly relevant component of the modern exposome [29]. Its residues are frequently detected in soil, water, and food, raising ongoing scientific and regulatory debates regarding their ecological impact and potential human health effects [30].

In contrast, mycotoxins represent an important class of natural pollutants. These toxic secondary metabolites commonly contaminate crops and food products. Climate change and suboptimal storage conditions further increase their prevalence. Chronic exposure to mycotoxins can be associated with carcinogenic, immunotoxic, and endocrine-disrupting effects [31].

A key challenge in exposome research is not only identifying individual hazards but understanding their combined, cumulative, and potentially synergistic effects over time. Recently published results demonstrated a close correlation between GLY and ZEA in endometrial carcinoma cases, highlighting the importance of synergistic effects among different environmental stress molecules in the context of endocrine disruption and oxidative stress effects [32]. This complexity complicates risk assessment and underscores the need for integrative monitoring approaches, improved regulatory frameworks, and preventive strategies in environmental and public health [33,34].

Taking the above into account, the GLY concentration measured in the ff was comparable to that measured in the serum. Based on our bibliographic research, this might be the first study to provide evidence of GLY detectability in human ff, thereby demonstrating that a widely used environmental contaminant can enter the close milieu of the developing oocyte and is only partially excluded by the follicular barrier. This observation indicates that GLY entry into the follicle is probably not effectively prevented, consistent with other reports of its presence in human matrices, including urine, blood, breast milk, and endometrium [4,14,32,35]. The findings and detected GLY concentrations in the present study are broadly consistent with a recently published human biomonitoring study reporting widespread but generally low-level GLY exposure in human populations [36]. No correlation was observed between serum and ff GLY levels. This may indicate that systemic exposure and follicular accumulation occur at different time points. Alternatively, GLY levels may be influenced by ovarian microenvironmental regulation. Accordingly, the lack of correlation between ffGLY levels and follicular protein levels may suggest that non-specific protein binding is not the primary determinant of follicular GLY levels. Importantly, no clear association was observed between GLY levels and ovarian function or standard ovarian indicators, including follicle count, number of retrieved oocytes, or embryo usability. GLY concentrations showed no correlation with commonly used *IVF* outcome parameters. This may imply that routine *IVF* outcome measurements are not sufficiently sensitive to early or subcellular biochemical alterations, such as exposure to low-dose environmental contaminants [24,37]. The negative trends observed in follicle, mature oocyte, and embryo numbers were not significant in the number of cases studied. Other studies have found a negative correlation between oocyte quantity and quality in larger case numbers [38,39]. On the other hand, oxidative stress markers, such as MDA, are more sensitive indicators of GLY-associated ovarian stress (within even the exposome framework, including mycotoxins). The elevated MDA levels reflect damage to polyunsaturated fatty acids, which are critical components of oocyte and granulosa cell membranes [24,40]. The positive association between ffGLY and MDA concentrations provides evidence that exposure to GLY or GBHs and lipid peroxidation are connected within the follicle [41,42,43]. The linear regression model demonstrated that GLY levels can explain almost one-third of the variability of MDA levels in the follicular environment. These findings are consistent with previous in vitro and in vivo studies that have addressed GLY-induced ROS generation, mitochondrial dysfunction, and lipid peroxidation in reproductive tissues [44,45]. Although antioxidants normally counteract oxidative stressors, such as ROS, during folliculogenesis, GLY-related oxidative stress may overwhelm these mechanisms. Vitamin C, E, and α-liponic acid (ALA) have been shown in prior studies to mitigate GLY-related oxidative damage to reproductive cells [46,47]. In our study, none of the antioxidant concentrations tested were correlated with GLY levels, suggesting that GLY might directly increase oxidative stress and not indirectly through increased stress or breakdown of the antioxidant system. The elevated MDA level indicates increased oxidative damage to cellular lipids, which may indirectly suggest a higher GLY level. This is supported by both animal and human studies showing a correlation between GLY and elevated urinary and tissue MDA concentrations [4,48].

The observed positive association between serum and ffGLY concentrations and E2 levels suggests that GLY acts as an endocrine disruptor at both systemic and follicular levels [49,50]. However, this finding contradicts previous literature data with epidemiological and experimental studies showing that GLY exposure suppresses steroidogenesis and alters the hypothalamic–pituitary–gonadal axis [14,41]. This potential trend requires validation in larger cohorts, because in the present study, significance disappeared after FDR correction. Further studies report alterations in additional enzymes involved in steroid hormone production, including StAR (steroidogenic acute regulatory protein), CYP11A1, and 3β-HSD, which are essential for cholesterol transport and conversion into active steroid hormones [51,52].

According to human epidemiological data, GLY exposure can reduce serum E2 levels even at current environmental levels, especially in adolescent girls [48,51]. In a study, activation of estrogen receptor alpha by constituents of glyphosate-based herbicides was demonstrated [51]. Unicsovics et al. reported that a direct link has been shown between E2 hormone-dependent high-grade endometrial carcinoma and glyphosate exposure [32]. The positive correlation we found, which contradicts previous results, may be due to the stimulation treatment in the *IVF* group we studied. However, GLY levels in serum and ff showed no association with the dose of the recombinant preparations. On the other hand, in the population we studied, we measured very low doses, well below the limit value. Low-dose GLY exposure has been shown to cause biological effects that often follow non-monotonic dose–response (NMDR) relationships, in which responses do not increase proportionally with dose and may even decrease at higher doses. Experimental studies have shown such patterns for both pure GLY and GLY-based formulations. For example, De Almeida et al. reported non-monotonic effects on cell viability and proliferation at low concentrations (starting at 10 µg/mL), suggesting that formulation components and ER status may influence toxicity and indicate potential endocrine-disrupting activity [53]. Recognition of NMDR relationships is therefore critical in toxicological risk assessment, as these responses may arise from mechanisms such as receptor desensitization, opposing receptor pathways, or feedback regulation. Ignoring NMDR dynamics may lead to underestimation of risks associated with low-level environmental exposures [54,55].

In contrast, GLY levels in ff show only a weak positive correlation with follicular E2. Alternatively, granulosa cells may increase estrogen production in response to oxidative stress [42]. GLY may interfere with E2 transport or clearance within the ff, leading to altered hormone fractions [11,14]. Although the correlation is statistically significant, its biological impact is limited due to confounding factors. Nevertheless, chronic low-level exposure to GLY in ff raises concern that it may impair follicular physiology over time in a non-deterministic manner [10].

The lack of association between GLY exposure and patient age, BMI, AMH, or gonadotropin levels may suggest that GLY-related effects and baseline endocrine markers are independent. This observation strengthens the theory that the effects of environmental toxicants may not be clinically assessed.

Folliculogenesis is highly sensitive to endocrine disruption. The many steps in the process by which primordial follicles develop into preovulatory follicles capable of ovulation are prone to being affected by endocrine harm [20]. Granulosa cells play a cardinal role in steroidogenesis and folliculogenesis. They express high levels of ERβ and are the primary site of ERβ-regulated gene expression in the ovary. Interruption of the ERβ signaling pathway may lead to arrested follicle development, consequently affecting oocyte maturation and ovulation [14,41]. Impaired ovarian function and follicular development caused by GLY and GBH exposure have been observed in numerous animal studies. Exposure to GBHs in rodent models has been associated with fewer primary and mature follicles, more atretic follicles, and histopathological changes, including interstitial fibrosis [11,56]. Multiple mechanisms at the cellular level have been attributed to GLY, including direct and indirect cytotoxicity in oocytes and granulosa cells [20,42,43,45]. In vitro studies using mouse and porcine oocyte models have demonstrated numerous harmful effects linked to GLY, including altered oocyte maturation, reduced rates of germinal vesicle breakdown and polar body extrusion, abnormal spindle morphology, and chromosomal misalignment [35,57]. Cao M et al. reported adverse reproductive effects in mice, affecting oocyte maturation and early embryonic development through mechanisms involving G protein-coupled estrogen receptor (GPER). The study also highlighted a strong protective effect of MT; its administration successfully mitigated the herbicide’s toxicity. MT rescued oocytes from damage by maintaining normal GPR30 receptor expression and activating the associated signaling pathway, the active and inactive Extracellular Signal-Regulated Kinase (pERK/ERK) pathway. These observations underscore the potential for endocrine-disrupting toxicity, which can occur even at low doses of environmentally relevant compounds, and demonstrate that MT is effective in mitigating this type of reproductive stress [58]. In our study, we found a slight but positive correlation between GLY and MT concentrations in the ff. However, the linear regression was not statistically significant, presumably due to the limited number of data points. Similarly, the protective function of MT mentioned earlier may also help in the follicular space against the endocrine and oxidative effects of GLY. Further studies are needed to analyze this in more detail. Direct evidence of GLY accumulation, or long-term presence in human tissues, is limited. However, its lipophilic properties and its documented presence in fluid samples suggest the potential for bioaccumulation in reproductive organs [4,7]. The proximity of the maturing oocyte to the ff poses a risk of impaired oocyte development and granulosa cell function, and thus fertility outcomes, even at low GLY levels [57,59]. Due to its chelating properties, GLY decreases intracellular zinc bioavailability and alters mitochondrial function and membrane potential. By generating ROS, GLY induces oxidative stress, which may trigger DNA breakage [49]. Moreover, the higher toxicity of Roundup molecules, including increased ROS generation and altered steroidogenesis, suggests that Roundup adjuvants may have their own biological activities and should be tested alone as active ingredients [60]. The observed inverse relationship between serum GLY and cortisol levels should be viewed with caution and interpreted similarly, as current experimental data linking GLY exposure to glucocorticoid regulation are limited and inconsistent. Animal studies suggest that increases in stress hormones are more common after exposure to GLY-based herbicides such as Roundup, whereas pure GLY typically causes little to no change in circulating corticosterone levels. Consistent with this, recent reviews indicate that GLY’s endocrine-disrupting effects have primarily been documented in sex steroid and thyroid hormone pathways, whereas its effects on glucocorticoid hormones, such as cortisol and corticosterone, remain poorly understood and rarely studied [8,61,62].

Long-term impacts and challenges of GLY and/or other exposome elements on female and male fertility and reproduction have been reported [14,44,56,63]. Several cohort and observational studies have reported associations between GLY exposure and pregnancy-related outcomes. Gerona et al. reported an association between early pregnancy glyphosate exposure and reduced fetal growth [63]. For example, biomonitoring research in U.S. populations has shown that higher urinary GLY levels in pregnant women are associated with shorter gestational length, suggesting potential effects on fetal development. Similarly, retrospective epidemiological data from rural populations in Canada indicated that preconception exposure to GLY was linked to an increased risk of late spontaneous abortions. Additional studies have documented higher GLY exposure levels among individuals living or working in agricultural settings, indicating occupational and environmental exposure pathways relevant to reproductive risk. Consequently, further large-scale, longitudinal studies integrating precise exposure assessment within the exposome framework are needed to clarify these associations.

### Limitations

The following limitations and challenges have been encountered during the study’s implementation and should also be considered when interpreting our results.

The Eurofins Abraxis ELISA method used in this study was primarily developed to analyze human serum samples for GLY; additionally, we tested ff. Although ff can be considered a transudate or exudate of serum and therefore has a biochemical makeup similar to that of blood serum, microenvironmental differences between the matrices may introduce analytical limitations. The manufacturer’s protocol also emphasizes that using matrices other than human serum requires thorough validation. However, the serum-like characteristics of ff, along with the high average recovery rate of 88.9% observed with serum samples, provide a sufficiently reliable basis for scientific evaluation. When interpreting the results, it is important to consider the method’s sensitivity; although ELISA is an extremely cost-effective and rapid screening tool for ff, future research might benefit from confirming the findings with mass spectrometry (e.g., LC-MS/MS) to fully rule out matrix effects and potential cross-reactivities, However, it is notable, that instrumental/chemical analyses (extraction, recovery, standardization, etc.) should also be set/validated according to properties of biological matrices applied. According to the manufacturer’s description, the ELISA reliably measures GLY levels in the matrix and does not cross-react with AMPA or others.

Although AMPA concentrations in the ff were not quantified in this study, this major metabolite may also influence hormonal, antioxidant, and oxidative stress processes. Analyzing this would be important in future studies. Therefore, total GLY-related exposure, even the acting exposome, is still incompletely characterized, the results reflect partial (parental) exposure only, and total glyphosate-related burden (GLY + AMPA) may be underestimated.

Additionally, we understand that our findings may be limited by the small sample size. The study was conducted on a relatively small cohort (*n* = 50), which limits statistical power and the generalizability of the findings, and increases the risk of both type I and type II errors.

Primarily, exploratory statistical analyses were applied, including multiple correlation tests. After FDR correction, the MDA-GLY association remained significant among the observed associations. However, the observed hormonal associations lost significance after corrections; they may represent trend-positive findings for future analyses. Additionally, a lack of detailed information regarding the specific vitamins consumed and patients’ dietary intake before sample collection might also be considered. For example, vitamin levels (vitamins C and E) and ALA were not assessed in this study; however, we plan to investigate these parameters in future research. It is possible that GLY measured in ff correlates more closely with the average serum GLY levels from the previous days or weeks, but no prior data support this hypothesis.

All participants underwent controlled ovarian stimulation, resulting in supraphysiological hormone levels by affecting the hypothalamic–pituitary–gonadal axis. This non-physiological condition, and possibly the sampling (pooled ff from different stages of follicles in both ovaries), may confound associations between GLY exposure and endocrine parameters, limiting extrapolation to natural cycles. However, ethically, it is nearly impossible to obtain permission for follicular fluid retrieval without a medical reason (and without stimulation).

## 5. Conclusions

In our study, we reported, probably for the first time, preliminary evidence of GLY presence (a potential component of the modern exposome) in human ff. We might confirm a link between MDA levels, which indicate oxidative stress, and GLY concentrations (in ng/mL), but it is possible that AMPA was not targeted in the study. This effect is not caused by depletion of the antioxidant system we examined. There was also a detectable relationship between serum and ffGLY levels in *IVF* women undergoing stimulation, supporting the endocrine-disrupting effect of GLY. Although raw follicle counts and stimulation results did not differ, the induced oxidative and hormonal effects may influence oocyte maturation and quality, thereby indirectly impacting fertility.

## Data Availability

The data presented in this study are available upon request from the corresponding author due to privacy and ethical considerations.

## Abbreviations

The following abbreviations are used in this manuscript:

Afs	Total Aflatoxins
ALA	α-liponic acid
AM1	Aflatoxin M1
AMH	Anti-Mullerian hormone
AMPA	aminomethylphosphonic acid
αZOL	alpha-Zearalenol
BMI	body mass index
CAT	Catalase
DHT	Dihydrotestosterone
DON	Deoxynivalenol
E2	Estradiol
EDC	Endocrine disrupting compound
ELISA	Enzyme-Linked Immunosorbent Assay
ER	Estrogen receptor
FB1	Fumonisin B1
FF	Follicular Fluid
FSH	Follicle-stimulating hormone
GBHs	Glyphosate-based herbicides
GLY	Glyphosate
IVF	In vitro fertilization
LC-MS/MS	Liquid chromatography-tandem mass spectrometry
LH	Luteinizing hormone
MII	Metaphase II oocyte
MT	Melatonin
NMDR	non-monotonic dose–response
OTA	Ochratoxin A
P4	Progesterone
ROS	Reactive Oxygen Species
SOD	Superoxid dismutase
TAOC	Total Antioxidant Capacity
ZEA	Zearalenone

## Appendix A

**Table A1:** Correlation data among serum and follicular GLY levels, biophysical parameters, hormone concentrations, examined mycotoxins, and antioxidants in serum and follicular fluid. It includes Spearman’s r and *p*-values (two-tailed), 95% confidence intervals, and the number of patients. The * indicates statistical significance. Ff: follicular, s: serum, GLY: Glyphosate, ZEA: Zearalenone, Afs: Aflatoxins, AM1: Aflatoxin M1, DON: Deoxynivalenol, αZOL: alpha-Zearalenol, OTA: Ochratoxin, FB1: Fumonisin B1, T2/HT2: T2/HT2 toxin, P4: progesterone, E2: estradiol, LH: luteotropic hormone, FSH: follicle-stimulating hormone, AMH: Anti-Mullerian hormone, DHT: Dihydrotestosterone, T: Testosterone, rFSH: recombinant FSH administered, MII: Metaphase II oocyte, TAOC: total antioxidant capacity, SOD: Superoxide-dismutase, MDA: malondialdehyde. * indicates significance *p* < 0.05, ** indicates significance *p* < 0.01; ns indicates non-significance.

	**ffGLY** **vs.** **ffZEA**	**ffGLY** **vs.** **ffAFs**	**ffGLY** **vs.** **ffAM1**	**ffGLY** **vs.** **ffDON**	**ffGLY** **vs.** **ffαZOL**	**ffGLY** **vs.** **ffOTA**	**ffGLY** **vs.** **ffFB1**	**ffGLY** **vs.** **ffT2/HT2**	**sGLY** **vs.** **s17-OH-P4**
**r**	−0.07520	−0.1136	0.1371	−0.1904	−0.03617	−0.07111	0.1685	−0.08070	0.1455
**95% confidence interval**	−0.3537 to 0.2156	−0.3872 to 0.1783	−0.1551 to 0.4073	−0.4519 to 0.1013	−0.3190 to 0.2526	−0.3501 to 0.2195	−0.1235 to 0.4338	−0.3586 to 0.2103	−0.1530 to 0.4198
***p*** **value**	0.6037	0.4321	0.3423	0.1854	0.8031	0.6236	0.2420	0.5774	0.3236
***p*** **value summary**	ns	ns	ns	ns	ns	ns	ns	ns	ns
**Number of XY Pairs**	50	50	50	50	50	50	50	50	50
	**ffGLY** **vs.** **LH**	**ffGLY** **vs.** **AMH**	**ffGLY** **vs.** **ffCortisol**	**ffGLY** **vs.** **ffE2**	**ffGLY** **vs.** **ff17OHP4**	**ffGLY** **vs.** **ffDHT**	**ffGLY** **vs.** **ffT**	**ffGLY** **vs.** **total rFSH**	**sGLY** **vs.** **sE2**
**r**	0.05147	−0.06786	0.1691	0.3090	0.3086	0.1074	0.1338	−0.02852	0.4521
**95% confidence interval**	−0.2382 to 0.3327	−0.3472 to 0.2226	−0.1230 to 0.4342	0.02192 to 0.5490	0.02149 to 0.5487	−0.2874 to 0.4711	−0.2485 to 0.4799	−0.3121 to 0.2597	0.1844 to 0.6573
***p*** **value**	0.7226	0.6396	0.2405	0.0307	0.0310	0.5864	0.4810	0.8441	0.0013
***p*** **value summary**	ns	ns	ns	*	*	ns	ns	ns	**
**Number of XY Pairs**	50	50	50	49	50	50	50	50	50
	**ffGLY** **vs.** **BMI**	**ffGLY** **vs.** **Age**	**ffGLY** **vs.** **MIItotal**	**ffGLY** **vs.** **No of Follicles**	**ffGLY** **vs.** **No of DominantFollicles**	**ffGLY** **vs.** **Useable embryos**	**ffGLY** **vs.** **total rFSH**	**sGly** **vs.** **LH**	**sGly** **vs.** **FSH**
**r**	0.1779	0.1352	0.06672	−0.02362	0.006667	−0.02972	−0.02852	0.1516	0.02600
**95% confidence interval**	−0.1141 to 0.4415	−0.1570 to 0.4057	−0.2237 to 0.3462	−0.3077 to 0.2643	−0.2800 to 0.2922	−0.3132 to 0.2586	−0.3121 to 0.2597	−0.1503 to 0.4276	−0.2712 to 0.3187
***p*** **value**	0.2165	0.3490	0.6453	0.8707	0.9633	0.8377	0.8441	0.3092	0.8623
***p*** **value summary**	ns	ns	ns	ns	ns	ns	ns	ns	ns
**Number of XY Pairs**	50	50	50	50	50	50	50	50	50
	**ffGLY** **vs.** **ffCAT**	**ffGLY** **vs.** **ffTAOC**	**ffGLY** **vs.** **ffSOD**	**ffGLY** **vs.** **ffMDA**	**ffGLY** **vs.** **ffMelatonin**	**sGLY** **vs.** **sMelatonin**	**sGLY** **vs.** **sCortisol**		
**r**	0.1768	0.1419	−0.1747	0.4487	0.2983	−0.08407	−0.3044		
**95% confidence interval**	−0.1152 to 0.4406	−0.1535 to 0.4139	−0.4389 to 0.1173	0.1865 to 0.6512	0.01012 to 0.5407	−0.3671 to 0.2132	−0.5478 to −0.01358		
***p*** **value**	0.2194	0.3309	0.2250	0.0011	0.0374	0.5700	0.0354		
***p*** **value summary**	ns	ns	ns	**	*	ns	*		
**Number of XY Pairs**	50	49	50	50	49	49	48		

## References

[1] Ombelet W. (2020). WHO fact sheet on infertility gives hope to millions of infertile couples worldwide. Facts Views Vis. Obgyn.

[2] Ma Y., He X., Qi K., Wang T., Qi Y., Cui L., Wang F., Song M. (2019). Effects of environmental contaminants on fertility and reproductive health. J. Environ. Sci..

[3] Yilmaz B., Terekeci H., Sandal S., Kelestimur F. (2020). Endocrine disrupting chemicals: Exposure, effects on human health, mechanism of action, models for testing and strategies for prevention. Rev. Endocr. Metab. Disord..

[4] Marino M., Mele E., Viggiano A., Nori S.L., Meccariello R., Santoro A. (2021). Pleiotropic Outcomes of Glyphosate Exposure: From Organ Damage to Effects on Inflammation, Cancer, Reproduction and Development. Int. J. Mol. Sci..

[5] Madani N.A., Carpenter D.O. (2022). Effects of glyphosate and glyphosate-based herbicides like Roundup™ on the mammalian nervous system: A review. Environ. Res..

[6] Lacroix R., Kurrasch D.M. (2023). Glyphosate Toxicity: In Vivo, In Vitro, and Epidemiological Evidence. Toxicol. Sci..

[7] Van Bruggen A.H.C., He M.M., Shin K., Mai V., Jeong K.C., Finckh M.R., Morris J.G. (2018). Environmental and health effects of the herbicide glyphosate. Sci. Total Environ..

[8] Agostini L.P., Dettogni R.S., Dos Reis R.S., Stur E., Dos Santos E.V.W., Ventorim D.P., Garcia F.M., Cardoso R.C., Graceli J.B., Louro I.D. (2020). Effects of glyphosate exposure on human health: Insights from epidemiological and in vitro studies. Sci. Total Environ..

[9] Ojelade B.S., Durowoju O.S., Adesoye P.O., Gibb S.W., Ekosse G.-I. (2022). Review of Glyphosate-Based Herbicide and Aminomethylphosphonic Acid (AMPA): Environmental and Health Impacts. Appl. Sci..

[10] Ingaramo P., Alarcón R., Muñoz-de-Toro M., Luque E.H. (2020). Are glyphosate and glyphosate-based herbicides endocrine disruptors that alter female fertility?. Mol. Cell. Endocrinol..

[11] Serra L., Estienne A., Vasseur C., Froment P., Dupont J. (2021). Review: Mechanisms of Glyphosate and Glyphosate-Based Herbicides Action in Female and Male Fertility in Humans and Animal Models. Cells.

[12] Galli F.S., Mollari M., Tassinari V., Alimonti C., Ubaldi A., Cuva C., Marcoccia D. (2024). Overview of human health effects related to glyphosate exposure. Front. Toxicol..

[13] Liu J.B., Li Z.F., Lu L., Wang Z.Y., Wang L. (2022). Glyphosate damages blood-testis barrier via NOX1-triggered oxidative stress in rats: Long-term exposure as a potential risk for male reproductive health. Environ. Int..

[14] Tajai P., Pruksakorn D., Chattipakorn S.C., Chattipakorn N., Shinlapawittayatorn K. (2023). Effects of glyphosate-based herbicides and glyphosate exposure on sex hormones and the reproductive system: From epidemiological evidence to mechanistic insights. Environ. Toxicol. Pharmacol..

[15] Revelli A., Delle Piane L., Casano S., Molinari E., Massobrio M., Rinaudo P. (2009). Follicular fluid content and oocyte quality: From single biochemical markers to metabolomics. Reprod. Biol. Endocrinol..

[16] Hussain T., Murtaza G., Metwally E., Kalhoro D.H., Kalhoro M.S., Rahu B.A., Sahito R.G.A., Yin Y., Yang H., Chughtai M.I. (2021). The Role of Oxidative Stress and Antioxidant Balance in Pregnancy. Mediat. Inflamm..

[17] Khazaei M., Aghaz F. (2017). Reactive Oxygen Species Generation and Use of Antioxidants during In Vitro Maturation of Oocytes. Int. J. Fertil. Steril..

[18] Ayala A., Muñoz M.F., Argüelles S. (2014). Lipid peroxidation: Production, metabolism, and signaling mechanisms of malondialdehyde and 4-hydroxy-2-nonenal. Oxid. Med. Cell Longev..

[19] Del Rio D., Stewart A.J., Pellegrini N. (2005). A review of recent studies on malondialdehyde as toxic molecule and biological marker of oxidative stress. Nutr. Metab. Cardiovasc. Dis..

[20] Yahfoufi Z.A., Bai D., Khan S.N., Chatzicharalampous C., Kohan-Ghadr H.R., Morris R.T., Abu-Soud H.M. (2020). Glyphosate Induces Metaphase II Oocyte Deterioration and Embryo Damage by Zinc Depletion and Overproduction of Reactive Oxygen Species. Toxicology.

[21] Lu L., Liu J.B., Wang J.Q., Lian C.Y., Wang Z.Y., Wang L. (2022). Glyphosate-induced mitochondrial reactive oxygen species overproduction activates parkin-dependent mitophagy to inhibit testosterone synthesis in mouse leydig cells. Environ. Pollut..

[22] Zinedine A., Soriano J.M., Moltó J.C., Mañes J. (2007). Review on the toxicity, occurrence, metabolism, detoxification, regulations and intake of zearalenone: An oestrogenic mycotoxin. Food Chem. Toxicol..

[23] Viegas S., Viegas C., Oppliger A. (2018). Occupational Exposure to Mycotoxins: Current Knowledge and Prospects. Ann. Work Expo. Health.

[24] Kaltsas A., Zikopoulos A., Moustakli E., Zachariou A., Tsirka G., Tsiampali C., Palapela N., Sofikitis N., Dimitriadis F. (2023). The Silent Threat to Women’s Fertility: Uncovering the Devastating Effects of Oxidative Stress. Antioxidants.

[25] Szentirmay A., Molnár Z., Plank P., Mézes M., Sajgó A., Martonos A., Buzder T., Sipos M., Hruby L., Szőke Z. (2024). The Potential Influence of the Presence of Mycotoxins in Human Follicular Fluid on Reproductive Outcomes. Toxins.

[26] Szőke Z., Ruff E., Plank P., Molnár Z., Hruby L., Szentirmay A., Unicsovics M., Csókay B., Varga K., Buzder T. (2025). Mycotoxin-Induced Oxidative Stress and Its Impact on Human Folliculogenesis: Examining the Link to Reproductive Health. Toxins.

[27] Wild C.P. (2005). Complementing the genome with an “exposome”: The outstanding challenge of environmental exposure measurement in molecular epidemiology. Cancer Epidemiol. Biomark. Prev..

[28] Gillezeau C., van Gerwen M., Shaffer R.M., Rana I., Zhang L., Sheppard L., Taioli E. (2019). The evidence of human exposure to glyphosate: A review. Environ. Health.

[29] Klátyik S., Simon G., Takács E., Oláh M., Zaller J.G., Antoniou M.N., Benbrook C., Mesnage R., Székács A. (2025). Toxicological concerns regarding glyphosate, its formulations, and co-formulants as environmental pollutants: A review of published studies from 2010 to 2025. Arch. Toxicol..

[30] Oummadi A., Menuet A., Méresse S., Laugeray A., Guillemin G., Mortaud S. (2023). The herbicides glyphosate and glufosinate and the cyanotoxin β-N-methylamino-l-alanine induce long-term motor disorders following postnatal exposure: The importance of prior asymptomatic maternal inflammatory sensitization. Front. Neurosci..

[31] Marín S., Cano-Sancho G., Sanchis V., Ramos A.J. (2018). The role of mycotoxins in the human exposome: Application of mycotoxin biomarkers in exposome-health studies. Food Chem. Toxicol..

[32] Unicsovics M., Szentirmay A., Fekécs G., Plank P., Molnár Z., Nagyéri G., Posta K., Ács N., Várbíró S., Sára L. (2026). Presence of glyphosate in endometrial cancer tissue: A cross-sectional study. Toxicol. Rep..

[33] Bennett J.W., Klich M. (2003). Mycotoxins. Clin. Microbiol. Rev..

[34] Tóth A.G., Nagy S.Á., Lakatos I., Solymosi N., Stágel A., Paholcsek M., Posta K., Gömbös P., Ferenczi S., Szőke Z. (2025). Impact of mycotoxins and glyphosate residue on the gut microbiome and resistome of European fallow deer. iScience.

[35] Wei X., Pan Y., Zhang Z., Cui J., Yin R., Li H., Qin J., Li A.J., Qiu R. (2024). Biomonitoring of glyphosate and aminomethylphosphonic acid: Current insights and future perspectives. J. Hazard. Mater..

[36] Connolly A., Koch H.M., Bury D., Koslitz S., Kolossa-Gehring M., Conrad A., Murawski A., McGrath J.A., Leahy M., Brüning T. (2022). A Human Biomonitoring Study Assessing Glyphosate and Aminomethylphosphonic Acid (AMPA) Exposures among Farm and Non-Farm Families. Toxics.

[37] Becatti M., Fucci R., Mannucci A., Barygina V., Mugnaini M., Criscuoli L., Giachini C., Bertocci F., Picone R., Emmi G. (2018). A Biochemical Approach to Detect Oxidative Stress in Infertile Women Undergoing Assisted Reproductive Technology Procedures. Int. J. Mol. Sci..

[38] Young A.S., Gennings C., Braselton M.E., Mullins C.E., Jariwala P., Liang D., Spencer J.B., Smith A.K., Hipp H., Shang W. (2025). Integrated chemical exposome-metabolome profiling of follicular fluid and associations with fertility outcomes during assisted reproduction. Environ. Int..

[39] Hallberg I., Plassmann M., Olovsson M., Holte J., Damdimopoulou P., Sjunnesson Y.C.B., Benskin J.P., Persson S. (2021). Suspect and non-target screening of ovarian follicular fluid and serum—Identification of anthropogenic chemicals and investigation of their association to fertility. Environ. Sci. Process. Impacts.

[40] Begum I.A. (2025). Oxidative stress: Oocyte quality and infertility. Reprod. Toxicol..

[41] Ren X., Li R., Liu J., Huang K., Wu S., Li Y., Li C. (2018). Effects of glyphosate on the ovarian function of pregnant mice, the secretion of hormones and the sex ratio of their fetuses. Environ. Pollut..

[42] Chitolina R., Nicola P., Sachett A., Bevilaqua F., Cunico L., Reginatto A., Bertoncello K., Marins K., Zanatta A.P., Medeiros M. (2023). Subacute exposure to Roundup^®^ changes steroidogenesis and gene expression of the glutathione-glutaredoxin system in rat ovaries: Implications for ovarian toxicity of this glyphosate-based herbicide. Toxicol. Appl. Pharmacol..

[43] Zhang J.W., Xu D.Q., Feng X.Z. (2019). The toxic effects and possible mechanisms of glyphosate on mouse oocytes. Chemosphere.

[44] de Liz Oliveira Cavalli V.L., Cattani D., Heinz Rieg C.E., Pierozan P., Zanatta L., Benedetti Parisotto E., Filho D.W., Silva F.R.M.B., Pessoa-Pureur R., Zamoner A. (2013). Roundup disrupts male reproductive functions by triggering calcium-mediated cell death in rat testis and Sertoli cells. Free Radic. Biol. Med..

[45] Bhardwaj J.K., Mittal M., Saraf P., Sharma S. (2022). Ameliorative potential of vitamin C and E against Roundup-glyphosate induced genotoxicity triggering apoptosis in caprine granulosa cells. Environ. Mol. Mutagen..

[46] Medithi S., Jonnalagadda P.R., Jee B. (2021). Predominant role of antioxidants in ameliorating the oxidative stress induced by pesticides. Arch. Environ. Occup. Health.

[47] Chang V.C., Andreotti G., Ospina M., Parks C.G., Liu D., Shearer J.J., Rothman N., Silverman D.T., Sandler D.P., Calafat A.M. (2023). Glyphosate exposure and urinary oxidative stress biomarkers in the Agricultural Health Study. J. Natl. Cancer Inst..

[48] Muñoz J.P., Bleak T.C., Calaf G.M. (2021). Glyphosate and the key characteristics of an endocrine disruptor: A review. Chemosphere.

[49] Sun X., Zhang H., Huang X., Yang D., Wu C., Liu H., Zhang L. (2024). Associations of glyphosate exposure and serum sex steroid hormones among 6–19-year-old children and adolescents. Ecotoxicol. Environ. Saf..

[50] Geier D.A., Geier M.R. (2023). Urine glyphosate exposure and serum sex hormone disruption within the 2013–2014 National Health and Nutrition Examination survey (NHANES). Chemosphere.

[51] Mesnage R., Defarge N., Spiroux de Vendômois J., Séralini G.E. (2015). Potential toxic effects of glyphosate and its commercial formulations below regulatory limits. Food Chem. Toxicol..

[52] Romano M.A., Romano R.M., Santos L.D., Wisniewski P., Campos D.A., de Souza P.B., Viau P., Bernardi M.M., Nunes M.T., de Oliveira C.A. (2012). Glyphosate impairs male offspring reproductive development by disrupting gonadotropin expression. Arch. Toxicol..

[53] De Almeida L.K.S., Pletschke B.I., Frost C.L. (2018). Moderate levels of glyphosate and its formulations vary in their cytotoxicity and genotoxicity in a whole blood model and in human cell lines with different estrogen receptor status. 3 Biotech.

[54] Lagarde F., Beausoleil C., Belcher S.M., Belzunces L.P., Emond C., Guerbet M., Rousselle C. (2015). Non-monotonic dose-response relationships and endocrine disruptors: A qualitative method of assessment. Environ. Health.

[55] van der Most M.A., Rietjens I.M.C.M., van den Brink N.W. (2024). Evaluating non-monotonic dose-response relationships in ecotoxicological risk assessment: A case study based on a systematic review of data on fluoxetine. Chemosphere.

[56] Milesi M.M., Lorenz V., Durando M., Rossetti M.F., Varayoud J. (2021). Glyphosate Herbicide: Reproductive Outcomes and Multigenerational Effects. Front. Endocrinol..

[57] Ospina M., Schütze A., Morales-Agudelo P., Vidal M., Wong L.Y., Calafat A.M. (2024). Temporal trends of exposure to the herbicide glyphosate in the United States (2013–2018): Data from the National Health and Nutrition Examination Survey. Chemosphere.

[58] Cao M., Wang Y., Yang F., Li J., Qin X. (2021). Melatonin rescues the reproductive toxicity of low-dose glyphosate-based herbicide during mouse oocyte maturation via the GPER signaling pathway. J. Pineal Res..

[59] Grau D., Grau N., Gascuel Q., Paroissin C., Stratonovitch C., Lairon D., Devault D.A., Di Cristofaro J. (2022). Quantifiable urine glyphosate levels detected in 99% of the French population, with higher values in men, in younger people, and in farmers. Environ. Sci. Pollut. Res. Int..

[60] Spinaci M., Nerozzi C., Tamanini Cl Bucci D., Galeati G. (2020). Glyphosate and its formulation Roundup impair pig oocyte maturation. Sci. Rep..

[61] Owagboriaye F., Dedeke G., Ademolu K., Olujimi O., Aladesida A., Adeleke M. (2019). Comparative studies on endogenic stress hormones, antioxidant, biochemical and hematological status of metabolic disturbance in albino rat exposed to roundup herbicide and its active ingredient glyphosate. Environ. Sci. Pollut. Res..

[62] de Araújo-Ramos A.T., Passoni M.T., Romano M.A., Romano R.M., Martino-Andrade A.J. (2021). Controversies on Endocrine and Reproductive Effects of Glyphosate and Glyphosate-Based Herbicides: A Mini-Review. Front. Endocrinol..

[63] Gerona R.R., Reiter J.L., Zakharevich I., Proctor C., Ying J., Mesnage R., Antoniou M., Winchester P.D. (2022). Glyphosate exposure in early pregnancy and reduced fetal growth: A prospective observational study of high-risk pregnancies. Environ. Health.

